# West Nile Virus Positive Blood Donation and Subsequent Entomological Investigation, Austria, 2014

**DOI:** 10.1371/journal.pone.0126381

**Published:** 2015-05-11

**Authors:** Jolanta Kolodziejek, Bernhard Seidel, Christof Jungbauer, Katharina Dimmel, Michael Kolodziejek, Ivo Rudolf, Zdenek Hubálek, Franz Allerberger, Norbert Nowotny

**Affiliations:** 1 Viral Zoonoses, Emerging and Vector-Borne Infections Group, Institute of Virology, University of Veterinary Medicine Vienna, Vienna, Austria; 2 Technical Office of Ecology and Landscape Assessment, Persenbeug, Austria; 3 Austrian Red Cross, Blood Service for Vienna, Lower Austria and Burgenland, Vienna, Austria; 4 Institute of Vertebrate Biology, Academy of Sciences of the Czech Republic, v.v.i., Brno, Czech Republic; 5 Department of Public Health, Austrian Agency for Health and Food Safety (AGES), Vienna, Austria; 6 Department of Microbiology and Immunology, College of Medicine and Health Sciences, Sultan Qaboos University, Muscat, Oman; National Institute of Health, ITALY

## Abstract

The detection of West Nile virus (WNV) nucleic acid in a blood donation from Vienna, Austria, as well as in *Culex pipiens* pupae and egg rafts, sampled close to the donor’s residence, is reported. Complete genomic sequences of the human- and mosquito-derived viruses were established, genetically compared and phylogenetically analyzed. The viruses were not identical, but closely related to each other and to recent Czech and Italian isolates, indicating co-circulation of related WNV strains within a confined geographic area. The detection of WNV in a blood donation originating from an area with low WNV prevalence in humans (only three serologically diagnosed cases between 2008 and 2014) is surprising and emphasizes the importance of WNV nucleic acid testing of blood donations even in such areas, along with active mosquito surveillance programs.

## Introduction

West Nile virus (WNV) is the most widespread flavivirus present in all continents except Antarctica. Up to 9 different genetic lineages have been described so far [1]; medically most important are lineages 1 and 2. WNV is maintained in a mosquito-bird transmission cycle, humans and horses are considered dead-end hosts. Most human infections are asymptomatic, however approximately 20% of cases develop a febrile illness with flu-like symptoms (West Nile fever, WNF) and less than 1% West Nile neuroinvasive disease (WNND), the latter associated with a mortality rate of about 10% [2]. The vast majority of patients acquire WNV infection through the bite of an infected mosquito, mainly of the genus *Culex* [3]. Other routes of transmission include blood transfusion, solid organ transplantation, congenital infection, and laboratory accidents [2]. Ticks may play an alternative role as vectors, especially in the introduction of WNV to new areas when attached to birds [4].

The first virologically confirmed WNV infections in Austria were reported for 2008 [5]. Goshawks proved to be especially vulnerable [5], [6]. In 2008, WNV nucleic acid was also detected in seven pools of adult female *Culex (Cx*.*) pipiens* mosquitoes [5]. Three autochthonous human WNV infections (two in 2009 and one in 2010; two cases of WNND and one case of WNF) were retrospectively diagnosed in the Greater Vienna area by specific serological testing [7].

In this paper we report on an acute WNV infection in a Viennese blood donor and the results of subsequent entomological investigations.

## Material and Methods

### Case record

As of June 2014, all blood donations originating from the province of Vienna are screened for WNV-RNA (PCR performed by the German Red Cross, Blood Service for Baden-Württemberg-Hessen, Frankfurt, Germany). A blood donation from 12 August 2014 tested positive for WNV-RNA. Serologically, the sample was WNV-IgM-positive and WNV-IgG-negative. Re-examination of the sample was performed at the Department of Virology of the Medical University of Vienna, the Austrian National Reference Laboratory for Flavivirus Infections. Following confirmation of the positive result, the Austrian Federal Office for Safety in Health Care (BASG), and the Austrian Federal Ministry of Health (BMG) were informed. Appropriate actions were taken according to the guidelines of the BMG from August 2014 (http://www.bmg.gv.at/cms/site2/attachments/2/7/5/CH1082/CMS1408527163324/westnilvirus_leitfaden_20140820.pdf), which correspond to the respective European legislation. On 19 August 2014 this case was also reported to ECDC (http://www.ecdc.europa.eu/en/healthtopics/west_nile_fever/West-Nile-fever-maps/pages/index.aspx).

### Ethics statement

Written informed consent to conduct this study was obtained from the affected blood donor. This investigation was part of a non-research public health emergency response and therefore exempt from the Institutional Review Board process of the City of Vienna. The Ethics Committee of Vienna as the competent Institutional Review Board has previously stated in writing (decision number EK 13-151-VK_NZ of July 1, 2013) that public health surveillance and public health emergency responses performed by the Austrian Agency for Health and Food Safety (AGES) are exempted from the Institutional Review Board process of the City of Vienna.

### Human WNV positive plasma sample

WNV-RNA positive plasma of the blood donor was provided by the Blood Service for Vienna, Lower Austria and Burgenland of the Austrian Red Cross. For PCR, pathogens in the sample were inactivated by immediately adding DNA/RNA Shield solution (Zymo Research, Irvine, USA) in the proportion 1:4 as described by [8], and stored at -20°C until further processing. The original plasma sample was independently investigated in the Czech laboratory for the presence of neutralizing antibodies against WNV strain Eg-101 by plaque-reduction neutralization test (PRNT) as described previously [9] and it was also used for virus isolation attempts (see below section “Virus isolation”).

### Mosquito collection

Following confirmation of the WNV-positive result of the human sample at the Medical University of Vienna, the BMG designated AGES to survey mosquitoes in close vicinity to the WNF patient's residence. The sampling started on 28 August 2014. The mosquitoes investigated in this study were collected on public land as part of the Austrian mosquito surveillance program. No additional permits to collect mosquitoes were required.

Trapping was performed using carbon-oxygen and attractants for pregnant female mosquitoes by BG-Sentinel traps (Biogents, Regensburg, Germany) and by manual exhauster. Because the field survey was carried out during a long-term rainfall period, when mosquitoes show low activity, the manual trapping was extended to aquatic mosquito stages. Collected mosquitoes were examined morphologically according to the identification key of Mohrig [10]. The specimens were sorted by species, their developmental stages, collection sites and dates, and pooled to a maximum number of 25 individuals.

Each mosquito pool was subsequently homogenized in an appropriate amount (300–700 μl, depending on the number of individuals) of cooled minimal essential medium (MEM, Gibco by Life Technologies, Grand Island, USA) supplemented with Earle's salts, non-essential amino acids (NEAA, 1%, Gibco by Life Technologies), and antibiotics. Homogenization was performed using an automatic TissueLyser II (Qiagen, Redwood City, USA) and Tungsten Carbide Beads 3 mm (Qiagen) followed by centrifugation at 10,000 x g for 5 min. All mosquito suspensions were stored at -80° C until processed.

### Sample screening

For nucleic acid extraction 140 μl of each sample was used. All extractions were performed with the QIAamp Viral RNA Mini Kit (Qiagen, USA), following the manufacturer’s instructions. For detection of both WNV lineages 1 and 2 in the samples, a RT-qPCR targeting the highly conserved 5´ non-coding region (NCR) was performed as described recently [4]. For confirmation and lineage determination a second RT-qPCR specific for WNV lineage 2 within the NS3 protein coding region was conducted as previously described [5].

### Virus isolation

The WNV positive samples were subjected to virus isolation attempts by intracerebral inoculation into suckling mouse brain (SMB), as described earlier [11]. The bacteriologically sterile SMB suspension (designated SMB_1_) was subsequently used for differential inoculation of adult (5–6 week old) ICR female specific-pathogen-free (SPF) mice. They were injected with 1% infectious SMB_1_ suspension intracerebrally (i.c., 0.04 ml, under anesthesia), 0.2 ml intraperitoneally (i.p.) and 0.2 ml subcutaneously (s.c.), respectively. Each of the three groups consisted of 4 mice.

All experiments with laboratory mice were conducted in Valtice, Czech Republic according to the Czech Animal Protection Act no. 246/1992. The protocols were approved by the Institutional and Central Care and Use Committees at the Academy of Sciences of the Czech Republic in Prague and by the Veterinary Service in Brno. The facility in Valtice is accredited by the Czech National Committee on Care and Use of Laboratory Animals (6630/2008-10001).

### RT-PCR, sequencing and sequence alignment

All positive samples and isolates were identified by various RT-PCRs targeting the complete WNV genome by employing published primer pairs specific for WNV lineage 2 [12], as well as self-designed primers specific for the Austrian WNV strains (primer sequences available upon request).

The RT-PCR assays were carried out using One Step RT-PCR Kit (Qiagen, USA) following the manufacturer’s instructions. Prior to sequencing, all specific amplification products were purified using PCR Kleen Spin Columns (BIO-RAD, Hercules, USA) following the manufacturer’s protocol. The purified PCR fragments were then premixed with the corresponding individual PCR primers (concentration of 2 μM each) in a volume of 15 μl. Sequencing in both directions was performed by Microsynth (http://www.microsynth.ch/). The obtained WNV sequences were manually verified and compiled to continuous sequences. Thereafter nucleotide sequences of the new WNVs were submitted to BLAST (http://www.ncbi.nlm.nih.gov/BLAST/) for further comparison with other WNV sequences deposited in GenBank databases. All complete genomic WNV sequences were downloaded individually in FASTA format. Sequences determined in this study were then compared to each other and to sequences from GenBank by using the Align Plus 4 program (Scientific & Education Software). Their nucleotide identities were determined.

### Polyprotein sequences and determination of pathogenicity and neuroinvasiveness markers

Translation of the new WNV sequences was carried out using the EMBOSS translation tools program (http://www.ebi.ac.uk/Tools/st/emboss_transeq/). Deduced entire polyprotein sequences were then compared to each other and to other polyprotein sequences deposited in GenBank databases employing the Align Plus 4 program (Scientific & Education Software) as described for the nucleotide sequences above.

To explore the pathogenicity and neuroinvasiveness markers of the newly determined WNV strains, predicted N-glycosylation sites of the relevant viral proteins E [13], [14] and NS1 [15] were analyzed using the program NetNGlyc 1.0 (http://www.cbs.dtu.dk/services/NetNGlyc/). Certain amino acids (P249 in NS3 and P250 in NS1) assumed to be associated with increased virulence were explored according to [16] and [17], respectively, summarized in [18]. Ability for neuroinvasiveness was furthermore investigated by laboratory mice experiments as described above (see section Virus isolation).

### Phylogenetic analysis

For phylogenetic analysis, nucleic acid sequences which encode the entire polyproteins but represent unique genomes were selected. For better resolution only WNV lineage 2 sequences were chosen, altogether 36 sequences (including sequences determined in this study). Synthetic constructs, incomplete (with gaps) and wrong (with Ns) sequences were excluded from the analysis.

Prior to phylogenetic analysis ClustalW multiple sequence alignments were conducted using BioEdit Sequence Alignment Editor Version 7.0.9.0. Several phylogenetic trees on both nucleotide and amino acid basis were constructed with the MEGA6 program [19] using the Maximum Composition Likelihood (MCL) and Kimura 2-parameter models of the Maximum Likelihood (ML) and Neighbor-Joining (NJ) methods. In each case bootstrap resampling analysis with 1,000 replicates was employed. The most likely tree was chosen.

### Intra- and inter-sequence groups distances

The MCL and p-distance algorithms of the MEGA6 were conducted for the determination of genetic distances within and between nucleotide and amino acid sequence groups, respectively. For this purpose sequence groups according to the clustering of the phylogenetic tree were defined. Furthermore, within the Central/Southern European cluster all new Austrian viruses and isolates and the nine most closely related viruses were subjected to detailed genetic distance analysis.

### Molecular determination of mosquito species

For confirmation of the morphologic typing, WNV-positive mosquito samples were investigated by a PCR assay within the mitochondrial 12S rDNA gene recommended for molecular determination of widely divergent arthropods [20]. For the genomic DNA-PCRs a Fast Cycling PCR Kit (Qiagen, USA) was applied. The specificity of PCR products was verified by sequencing, as mentioned above (see section RT-PCR, sequencing and sequence alignment).

### GenBank accession numbers

The newly described Austrian complete WNV sequences are available from GenBank under accession numbers KP109691 (WNV lin. 2, blood donor/Vienna/2014) and KP109692 (WNV lin. 2, *Cx*. *pipiens*/Vienna/2014). The 388 bp long 12S rDNA gene sequence of the WNV-positive *Cx*. *pipiens* is available in GenBank under accession number KP109693.

## Results

### Blood donor

The blood donor was a 44-year-old Viennese female. Three days after her blood donation on 12 August 2014 she developed myalgia, and later a generalized maculopapular rash. Her travel history outside Austria only revealed a trip to Barcelona, Spain, in February 2014. She remembered numerous mosquito bites in the weeks before blood donation when gardening at her home in the city of Vienna.

### Human plasma sample

The neutralizing antibody titer of the human plasma sample against WNV by PRNT was 1:40.

The positive WNV-RNA results of this sample were confirmed by the two RT-qPCR assays described above, revealing WNV lineage 2. In addition WNV from the original plasma sample of the blood donor was successfully isolated in suckling mice: one of five mice died on day 6 post inoculation (p.i.). The brain suspension of the dead mouse SMB_1_ was further passaged. In the next passage all of the 11 inoculated mice died on days 3 and 4 p.i. with neurological symptoms.

Further experimental inoculation of 1% centrifuged mouse brain homogenate of the SMB_1_ plasma isolate into 12 adult SPF mice by 3 different routes caused death of all mice inoculated. I.c. inoculated mice died after 6–7 days (average survival time AST = 6.7 days), i.p. inoculated mice died after 6–11 days (AST = 8.5 days), and s.c. inoculated mice died after 10 and more days. The obtained isolates are subsequently named SPF i.c. and SPF i.p.

The presence of WNV-RNA in the plasma isolates SMB_1_, SPF i.c. and SPF i.p. was confirmed by RT-qPCR.

By a series of specific RT-PCRs and subsequent sequencing the entire WNV genomes of the original plasma sample as well as of its isolates SMB_1_, SPF i.c. and SPF i.p. were determined and compared to each other. Mutations were analyzed.

### Mosquitoes

A total of 603 mosquitoes (when one egg raft was counted as one individual mosquito) were trapped in Vienna between 28 August and 10 September 2014, and assigned to 45 pools (Table 1).

**Table 1:** Mosquitoes collected in the city of Vienna between 28 August and 10 September 2014.

No.	Location	Date	Species	Stage	Quantity	MEM [μl]
1	BFH	28.08.14	*Cx*. *pipiens*	L	20	600
2	BFH	28.08.14	*Cx*. *pipiens*	L	2	300
3	BFH	28.08.14	*Cx*. *pipiens*	L	20	600
4	BFH	29.08.14	*Cx*. *pipiens*	L	16	500
5	BFH	29.08.14	*Cx*. *pipiens*	L	20	600
6	BFH	29.08.14	*Cx*. *pipiens*	L	21	600
7	BFH	30.08.14	*Cx*. *pipiens*	E	5	300
8	BFH	30.08.14	*Cx*. *pipiens*	L	18	600
9	FH Ottakring	08.09.14	*Cx*. *pipiens* (+)	E	2	300
10	FH Ottakring	08.09.14	*Cx*. *pipiens*	E	2	300
11	FH Ottakring	08.09.14	*Cx*. *pipiens*	L	15	500
12	FH Ottakring	08.09.14	*Cx*. *pipiens*	L	14	500
13	FH Ottakring	08.09.14	*Cx*. *pipiens*	P	15	500
14	FH Ottakring	08.09.14	*Cx*. *pipiens*	P	15	500
15	FH Ottakring	08.09.14	*Cx*. *pipiens*	P	15	500
16	FH Ottakring	08.09.14	*Cx*. *pipiens*	L	15	500
17	FH Ottakring	08.09.14	*Cx*. *pipiens*	L	15	500
18	FH Ottakring	08.09.14	*Cx*. *pipiens*	L	15	500
19	FH Ottakring	08.09.14	*Cx*. *pipiens*	P	15	500
20	FH Ottakring	08.09.14	*Cx*. *pipiens* (+)	P	15	500
21	FH Ottakring	08.09.14	*Cx*. *pipiens*	P	15	500
22	FH Ottakring	08.09.14	*Cx*. *pipiens*	P	15	500
23	FH Ottakring	08.09.14	*Cx*. *pipiens*	P	25	700
24	FH Ottakring	08.09.14	*Cx*. *pipiens*	L	15	500
25	FH Ottakring	08.09.14	*Cx*. *pipiens*	L	15	500
26	FH Ottakring	08.09.14	*Cx*. *pipiens*	L	15	500
27	FH Ottakring	08.09.14	*Cx*. *pipiens*	L	15	500
28	Lainz Zoo	29.08.14	*Cx*. *pipiens*	E	2	300
29	Lainz Zoo	30.08.14	*Cx*. *pipiens*	L	20	600
30	Lainz Zoo	30.08.14	*Cx*. *pipiens*	L	20	600
31	Lainz Zoo	30.08.14	*Cx*. *pipiens*	L	20	600
32	Lainz Zoo	30.08.14	*Cx*. *pipiens*	L	20	600
33	Lainz Zoo	30.08.14	*Cx*. *pipiens*	L	20	600
34	Lobau Polzer	06.09.14	*An*. *maculipennis*	A	2	300
35	Schönbrunn	02.09.14	*Cx*. *pipiens*	L	15	500
36	Schönbrunn	02.09.14	*Cx*. *pipiens*	L	15	500
37	Schönbrunn	02.09.14	*Cx*. *pipiens*	L	20	600
38	Schönbrunn	02.09.14	*Cx*. *pipiens*	L	20	600
39	Schönbrunn	02.09.14	*An*. *maculipennis*	A	4	300
40	Schönbrunn	02.09.14	*Cs*. *annulata*	A	1	300
41	Schönbrunn	02.09.14	*Cx*. *pipiens*	A	1	300
42	Schönbrunn	02.09.14	*An*. *plumbeus*	A	1	300
43	Schönbrunn	03.09.14	*Cx*. *pipiens*	A	2	300
44	Schönbrunn	10.09.14	*Cx*. *pipiens*	A	15	500
45	Schönbrunn	10.09.14	*Cx*. *pipiens*	A	15	500

The two WNV-positive samples are marked with (+). Abbreviations used: BFH, Baumgarten Cemetery; FH Ottakring, Ottakring Cemetery; *An*., *Anopheles*; *Cs*., *Culiseta*; *Cx*., *Culex*; A, adults; E, egg rafts; L, larvae; P, pupae; MEM, minimal essential medium.

Mosquito trapping was performed in five locations in Vienna, near to the patient’s home: Baumgarten cemetery (48°12'07.7"N / 16°16'44.8"E; 8 pools, n = 122), Lainz zoo (48°12'09.3"N / 16°13'57.0"E; 6 pools, n = 102), Schönbrunn (48°10'55.5"N / 16°19'20.5"E; 11 pools, n = 109), Lobau Polzer (48°11'40.6"N / 16°28' 06.7"E; 1 pool, n = 2), and Ottakring cemetery (48°12'52.6"N / 16°17'58.0"E; 19 pools, n = 268).

A total of 4 different mosquito species were morphologically identified: *Cx*. *pipiens* (41 pools, n = 595), *Anopheles (An*.*) maculipennis* group (2 pools, n = 6), *Culiseta (Cs*.*) annulata* (1 pool, n = 1), and *An*. *plumbeus* (1 pool, n = 1); thus *Cx*. *pipiens* represented 98.67% of our mosquito collection. All developmental stages were collected: egg rafts (4 pools, n = 11), larvae (25 pools, n = 421), pupae (8 pools, n = 130) and adult mosquitoes (8 pools, n = 41); therefore non-adult mosquitoes represented 93.20% of the mosquito collection.

Out of the 45 mosquito pools investigated, two pools proved positive for WNV lineage 2: one pool of 15 *Cx*. *pipiens* pupae, and one pool of two *Cx*. *pipiens* egg rafts, both collected on 08 September 2014 near Ottakring cemetery, 500m distance to the patient's home. Molecular determination confirmed the mosquito species.

The relative abundance of WNV in the investigated mosquito pools was 4.44%. The minimal infection rate (MIR) for all mosquitoes collected was 0.332 (converted to MIR per 1,000 mosquitoes, 0.551).

Virus isolation attempt on the *Cx*. *pipiens* pupae suspension failed; no suckling mouse of 10 inoculated died. The *Cx*. *pipiens* egg rafts suspension was not subjected to virus isolation due to unsufficient quantity and quality of the sample.

Out of the mosquito pool containing 15 *Cx*. *pipiens* pupae a complete WNV genomic sequence was determined, while only a few partial sequences (approx. 20% of the genome) could be obtained from the sample which contained two *Cx*. *pipiens* egg rafts due to above mentioned reason. However, their corresponding sequences were 100% identical to each other.

### Comparison of human- and mosquito-derived WNV strains

While the viral loads in the human plasma sample and in the mosquito pool consisting of 15 pupae were identical (quantification cycle [Cq] for both = 32), the quantity of viral RNA was less in the sample containing the two egg rafts (Cq = 37). In the SMB_1_, SPF i.p. and SPF i.c. plasma isolates 10^6^-, 10^7^- and 10^8^-fold more viral RNA was detected than in the original plasma sample.

WNV genomes determined in this study were 10,988 nucleotides in length. The deduced complete polyproteins of all strains consisted of 3,434 amino acids, along which the three known flaviviral structural and eight non-structural proteins could be defined. The lengths of the corresponding individual proteins were: 123 (C), 167 (prM/M), 501 (E), 352 (NS1), 231 (NS2A), 131 (NS2B), 619 (NS3), 122 (NS4A), 27 (2K), 256 (NS4B), and 905 (NS5), respectively.

**Fig 1:**
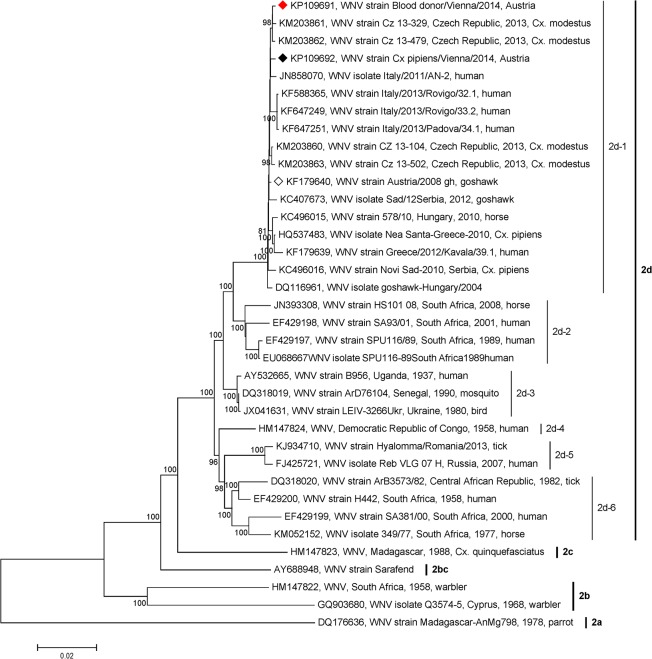
Phylogenetic tree of 36 West Nile virus lineage 2 full length polyprotein-coding nucleotide sequences. The sequences determined in this study are marked with a red diamond (Viennese blood donor-derived WNV) and a black diamond (Viennese *Culex pipiens*-derived WNV), respectively. The Austrian goshawk-derived sequence from 2008 is marked with a contoured diamond. Five major clades and six clusters among clade 2d are indicated. Group 2d-1 contains the Central/Southern European viruses including Austrian strains, and group 2d-5 consists of the Eastern European WNVs. The phylogenetic tree was constructed using the NJ method with MCL algorithm of MEGA6 [19] with 1,000-fold bootstrap analysis. GenBank accession numbers, strain names, and (if known) species, countries of origins and years of isolations are indicated at the branches. Supporting bootstrap values >80% (the percentage of replicates in the bootstrap analysis) are displayed next to the nodes. The horizontal scale bar indicates genetic distances (here 2% nucleotide sequence divergence).

The Austrian human plasma-derived WNV showed the least nucleotide and amino acid divergences (0.2% and 0.1%, respectively) to WNV strains Cz 13–329 and Cz 13–479, both isolated in 2013 from *Cx*. *modestus* mosquitoes in the Czech Republic, belonging to the Central/Southern European WNV lineage 2 group (Table 2A, Fig 1). The Austrian mosquito-derived WNV nucleotide sequence exhibited the least nucleotide (0.3%) and amino acid (0.1%) genetic distance to the Czech strain Cz-104 and the Austrian goshawk-derived WNV (Table 2A, Fig 1). Detailed nucleotide and amino acid distances over the Austrian strains (including SMB_1_ plasma isolate) and their nine relatives defined according to the clustering in the phylogenetic tree (Fig 1) are indicated in Table 2A.

**Table 2:** Estimates of evolutionary pairwise distances A. over the Austrian strains (including the SMB_1_ plasma isolate) and their nine closest relatives, B. five major groups (clades), and C. six minor groups (clusters) among clade 2d, all defined according to the clustering in the phylogenetic tree (Fig 1).

A												
	At-bd	SMB_1_	Cz 329	Cz 479	At-Cx	It AN2	It 32.1	It 33.2	It 34.1	Cz 104	Cz 502	At-gh
**At-bd**	-	0.0003	0.0009	0.0015	0.0015	0.0026	0.0023	0.0026	0.0035	0.0012	0.0018	0.0012
**SMB**_**1**_	0.0001	-	0.0012	0.0018	0.0018	0.0029	0.0026	0.0029	0.0038	0.0015	0.0020	0.0015
**Cz 329**	0.0020	0.0021	-	0.0012	0.0012	0.0023	0.0026	0.0023	0.0032	0.0009	0.0015	0.0009
**Cz.479**	0.0018	0.0019	0.0021	-	0.0018	0.0029	0.0032	0.0029	0.0038	0.0015	0.0020	0.0015
**At-Cx**	0.0032	0.0033	0.0035	0.0031	-	0.0023	0.0026	0.0023	0.0032	0.0009	0.0015	0.0012
**It AN2**	0.0036	0.0037	0.0037	0.0035	0.0034	-	0.0038	0.0035	0.0044	0.0020	0.0026	0.0023
**It 32.1**	0.0042	0.0043	0.0047	0.0043	0.0043	0.0047	-	0.0003	0.0012	0.0023	0.0029	0.0026
**It 33.2**	0.0041	0.0042	0.0044	0.0040	0.0040	0.0044	0.0005	-	0.0009	0.0020	0.0026	0.0023
**It 34.1**	0.0046	0.0047	0.0049	0.0045	0.0045	0.0049	0.0010	0.0007	-	0.0029	0.0035	0.0032
**Cz 104**	0.0031	0.0032	0.0032	0.0030	0.0030	0.0034	0.0040	0.0037	0.0042	-	0.0006	0.0009
**Cz 502**	0.0037	0.0038	0.0038	0.0036	0.0036	0.0040	0.0046	0.0043	0.0048	0.0014	-	0.0015
**At-gh**	0.0028	0.0029	0.0029	0.0027	0.0027	0.0029	0.0037	0.0034	0.0039	0.0020	0.0026	-
**B**												
	**2d**	**2c**	**2bc**	**2b**	**2a**							
**2d**	-	0.0114	0.0121	0.0227	0.0335							
**2c**	0.0689	-	0.0125	0.0226	0.0332							
**2bc**	0.0744	0.0813	-	0.0239	0.0344							
**2b**	0.1204	0.1255	0.0194	-	0.0401							
**2a**	0.1988	0.2045	0.1999	0.2060	-							
**C**												
	**2d-1**	**2d-2**	**2d-3**	**2d-4**	**2d-5**	**2d-6**						
**2d-1**	-	0.0063	0.0057	0.0054	0.0061	0.0067						
**2d-2**	0.0257	-	0.0053	0.0050	0.0058	0.0063						
**2d-3**	0.0238	0.0202	-	0.0042	0.0052	0.0058						
**2d-4**	0.0353	0.0317	0.0194	-	0.0050	0.0053						
**2d-5**	0.0410	0.0389	0.0264	0.0313	-	0.0055						
**2d-6**	0.0394	0.0356	0.0240	0.0284	0.0311	-						

The average numbers of substitutions per site between nucleotide and amino acid sequences are indicated below and above the diagonal, respectively. Estimation of evolutionary distances was conducted in MEGA6 [19] using the MCL and p-distance algorithms for nucleotide and amino acid sequences, respectively. Group 2d-1 contains the Central/Southern European viruses including Austrian strains, and group 2d-5 consists of the Eastern European WNVs.

Abbreviations used: At-bd = strain Blood donor/Vienna/2014Austria (KP109691), SMB_1_ = first passage of the suckling mouse brain isolate from the Austrian blood donor’s plasma, Cz 329 = strain Cz 13–329 (KM203861), Cz 479 = strain Cz 13–479 (KM203862), At-Cx = strain Cx pipiens/Vienna/2014Austria (KP109692), It AN2 = isolate Italy/2011/AN-2 (JN858070), It 32.1 = strain Italy/2013/Rovigo/32.1 (KF588365), It 33.2 = strain Italy/2013/Rovigo/33.2 (KF647249), It 34.1 = strain Italy/2013/Padova/34.1 (KF647251), Cz 104 = s train CZ 13–104 (KM203860), Cz 502 = strain Cz 13–502 (KM203863), At-gh = WNV strain Austria/2008_goshawk (KF179640).

The comparison of the entire polyprotein sequences of the Viennese human- and mosquito-derived WNV strains revealed 36 nucleotide (genetic distance 0.003) and five amino acid substitutions (genetic distance 0.001) which were found among the E (A 159 T, T 424 A) and NS5 (R 314 K, R 576 Q, K 638 E) genes. All remaining genes exhibited identical amino acid sequences.

The comparison of the entire polyprotein sequences of the Viennese human WNV strain and its isolate SMB_1_ revealed only one nucleotide substitution (T to A at position 1332 of the entire polyprotein coding nucleotide sequence), resulting in amino acid change asparagine (N) to lysine (K) at position 154 within the E gene. The subsequent WNV plasma passages SPF i.c. and SPF i.p exhibited 100% identity to isolate SMB_1_.

Markers associated with increased pathogenicity and neuroinvasiveness could be identified in all Austrian WNV strains and isolates: three potential N-glycosylation sites at positions N-130, N-175, and N-207 [15] and proline at position 250 of the NS1 gene [17]. Proline at position 249 of the NS3 gene responsible for higher strain virulence [16] was, however, not identified in any of the Austrian strains. At this position histidine was determined. Furthermore, while in both the original human plasma- and mosquito-derived sequences N-glycosylation motif NYS at positions 154–156 of the E protein were identified [13], [14], it was no longer found in the sequences obtained from SMB_1_, SPF i.c. and SPF i.p. passages of the plasma isolate. As mentioned above, at position 154, lysine instead of asparagine was identified. Experimental infection of adult SPF mice with mouse brain homogenate SMB_1_ resulted in 100% mortality.

### Phylogenetic analysis and genetic distances

The phylogenetic tree based on nucleotide sequences coding for entire polyproteins constructed by the NJ method and MCL model of the MEGA6 program support a clear division of the WNV lineage 2 strains into the recently described four clades (2a-2d) [21]. While clades 2a and 2c consist of only one strain each (both from Madagascar), clade 2b is composed of two WNV strains, one from South Africa 1958 and the other from Cyprus 1968 (Fig 1). Central/Southern European strains (cluster 2d-1) cluster within the largest clade 2d together with Eastern European strains (cluster 2d-5) and several viruses mostly from Africa, isolated between 1937 and 2008 (clusters 2d-2, 2d-3, 2d-4 and 2d-6; Fig 1). As strain Sarafend was not included in the study by Mac Mullen [21], we denoted it clade 2bc due to its position between clades 2b and 2c. The division into the above clades and clusters was confirmed using the ML method of MEGA6 (data not shown). Similar clustering was also obtained by analyzing the corresponding entire polyprotein sequences (data not shown).

In the phylogenetic analysis both newly determined Austrian WNV strains cluster next to recently isolated Czech and Italian strains as well as to the 2008 goshawk-derived Austrian strain and other Central/Southern European lineage 2 strains, sorted by temporal sequence of WNV detections from 2004 to 2014 (Fig 1, cluster 2d-1).

The nucleotide and amino acid genetic distances over all sequence pairs between major sequence groups (clades) 2a-2d and 2bc as well as between clusters 2d-1 and 2d-6 are shown in Table 2B and 2C, respectively. The least distances on both nucleotide and amino acid levels were calculated between clusters 2d-3 and 2d-4 (0.0194, 0.0042), and the maximum distances between clades 2a and 2b (0.2060, 0.0401). The genetic distance between the Central/Sothern (2d-1) and Eastern (2d-5) European lineage 2 sequence groups was calculated with 0.0410 (nucleotides) and 0.0061 (amino acids). The numbers of differences per site over all sequence pairs within clades 2b and 2d were 0.0983 and 0.0228 for nucleotides and 0.0242 and 0.0050 for amino acids, respectively. The numbers of differences per site over all sequence pairs within clusters 2d-1, 2d-2, 2d-3, 2d-5 and 2d-6 were 0.0045, 0.0124, 0.0016, 0.0054 and 0.0215 for nucleotides, and 0.0027, 0.0046, 0.0021, 0.0017 and 0.0055 for amino acids, respectively.

## Discussion

The potential for WNV transmission by blood transfusion during the acute phase of infection, when infected individuals are asymptomatic but viremic, was first recognized in the United States [22]. Soon thereafter WNV transmission by organ transplantation was reported [23]. Twenty-three confirmed cases of WNV transmission by blood or blood components were documented in 2002 [23], resulting in the implementation of a stringent blood safety monitoring system in the U.S. ([24], http://www.cdc.gov/westnile/resources/pdfs/wnvguidelines.pdf).

Independent introductions of two different WNV lineage 2 strains from Africa to Europe occurred recently: the first strain was introduced to Central Europe (South-Eastern Hungary) in or before 2004 [12], dispersed all over Hungary and the eastern part of Austria in 2008 [5], [6], has spread in the following years via the Balkan Peninsula [25] to Southern European countries [26], [27], and arrived to the Czech Republic in 2013 [11], while the other strain emerged in Eastern Europe (Russia and Romania) since 2007 and 2010, respectively [28], [29], [4]. Both strains have been responsible for several outbreaks in the EU with 128 autochthonous cases reported in 2011, 242 in 2012, and 228 in 2013 (http://www.ecdc.europa.eu/en/healthtopics/west_nile_fever/West-Nile-fever-maps/Pages/historical-data.aspx), with case fatality rates of 8% in Romania in 2010 [29], 15% in Greece between 2010 and 2011 [30] and about 10% in Italy between 2008 and 2012 [31]. In 2014, apart from the Austrian case, 73 further human cases of WNV infection have been reported in the EU and 136 in neighboring countries (http://www.ecdc.europa.eu/en/healthtopics/west_nile_fever/West-Nile-fever-maps/pages/index.aspx).

Nucleic acid testing (NAT) of blood supplies was initiated in Italy following the first human cases of WNND during the 2008 outbreak [31]. Since then, a total of 71 human cases of WNND have been reported in Italy until 2012 and 26 WNV positive blood donations could be detected by NAT [31].

In order to ensure safety and quality of the blood transfusion chain in Europe, a guidance was introduced at the European Union level (http://ec.europa.eu/health/blood_tissues_organs/docs/wnv_preparedness_plan_2012.pdf), which has been continuously updated.

As the incubation period of WNF is typically between 2 and 15 days, the use of NAT techniques has provided an opportunity to diagnose WNV in patients prior to the production of specific IgM antibodies, as the circulation of detectable levels of WNV RNA in blood occurs, on average, 4 days prior to the first detection of IgM antibodies [32]. WNV RNA generally became undetectable after 13.2 days [33], thus, the detection of both IgM antibodies and viral RNA of the Viennese patient indicates very recent infection.

As the Viennese blood donor was not abroad in the last 6 months before infection, it was considered an autochthonous case. This is supported by the identification of WNV-positive *Cx*. *pipiens* mosquitoes collected in the residential area of the blood donor. The genetic differences between the two virus strains are not surprising, as the co-circulation of similar, but not identical WNV strains in restricted areas, has been reported previously [11].

The Austrian WNV strains investigated here carry only a few suspected neuroinvasiveness and pathogenicity markers. Interestingly, the highly conserved N-glycosylation site N-154 of the E gene, which has been associated with significant human outbreaks including the North American epidemic [13] and which was initially identified in both the Austrian human plasma and mosquito pool, mutated to lysine (K-154) during the virus isolation process of the plasma sample in suckling and adult mice. Despite (or because) of lack of glycosylation of this site, the WNV plasma isolate turned out to be highly neuroinvasive for adult mice. Such a mutation was also observed among the Russian WNV strains isolated in Volgograd 1999 from human brains indicating their high neuroinvasivness and pathogenicity (GenBank acc. nos. AY277252 and AF317203) [21]. Studies in mice revealed that—while both non- and E-glycosylated WNV strains were equally neurovirulent—the latter were more neuroinvasive [13]. The WNV strain investigated in our study had fortunately caused only mild febrile illness in the Viennese patient, possibly related to the comparatively young age of the patient. In addition, we do not expect that the neuroinvasive properties of the plasma isolate were due to the presence of lysine at position E-154 or due to the lack of the N-154 glycan, however this exceptional point mutation observed in the present study requires further analysis.

Despite the limited number of mosquitoes collected for this study (45 pools), WNV was detected at least in two individuals. Compared to recent Czech and Hungarian studies, in which WNV-positive mosquitoes were found in four of 650 pools [11] and in three of 645 pools [34], respectively, the MIR in our mosquito collection seems to be relatively high. A possible explanation for this phenomenon could be the fact that more than 90% of mosquitoes collected in Vienna represented non-adult individuals, including egg rafts, pupae and larvae, which may be progenies of a few infected adult mosquitoes. Most published WNV detections in mosquitoes were in adult individuals only, and reports of detections in different developmental stages of mosquitoes are scarce [35].

While over 65 mosquito species have been implicated in the transmission of WNV, the principal mosquito vector species are those belonging to the genus *Culex* [3], [33], Fig 1. Although *Cx*. *pipiens* are essentially ornithophilic mosquitoes, their blood meal may be taken from mammals, including humans [36]. Hence, infected females may contribute to avian and human infections by horizontal transmission of the virus during their blood feedings, but also to vertical virus transmission [37]. Vertically infected *Cx*. *pipiens* that entered diapause in late autumn are able to initiate infection in the following spring [37]. Thus, detection of WNV-RNA in egg rafts and pupae deposited by infected females during late summer or fall may provide evidence for the vertical passage of WNV to overwintering cohorts.

The detection of WNV in a blood donation originating from an area with rather low WNV prevalence in humans is surprising and emphasizes the importance of NAT screening of blood donations even in areas of low WNV prevalence, along with active mosquito surveillance programs.
